# Temperate phage-antibiotic synergy is widespread—extending to *Pseudomonas*—but varies by phage, host strain, and antibiotic pairing

**DOI:** 10.1128/mbio.02559-24

**Published:** 2024-12-20

**Authors:** Rabia Fatima, Alexander P. Hynes

**Affiliations:** 1 Department of Biochemistry and Biomedical Sciences, McMaster University, Hamilton, Ontario, Canada; 2 Department of Medicine, McMaster University, Hamilton, Ontario, Canada; 3 Farncombe Family Digestive Health Research Institute, McMaster University, Hamilton, Ontario, Canada; 4 Michael G. DeGroote Institute for Infectious Disease Research, McMaster University, Hamilton, Ontario, Canada; University of Nebraska-Lincoln, Lincoln, Nebraska, USA

**Keywords:** bacteriophages, antibiotics, temperate phages, phage-antibiotic synergy, *Pseudomonas aeruginosa*, clinical isolates

## Abstract

Bacteriophages (phages) are bacterial-specific viruses that can be used alone or with antibiotics to reduce bacterial load. Most phages are unsuitable for therapy because they are “temperate” and can integrate into the host genome, forming a lysogen that is protected from subsequent phage infections. However, integrated phages can be awakened by stressors such as antibiotics. Supported by this interaction, here we explore the potential use of combined temperate phage and antibiotic against the multi-drug-resistant pathogen, *Pseudomonas aeruginosa*. In all, thirty-nine temperate phages were isolated from clinical strains, and a subset was screened for synergy with six antibiotics (ciprofloxacin, levofloxacin, meropenem, piperacillin, tobramycin, and polymyxin B), using checkerboard assays. Interestingly, our screen identified phages that can synergize with each antibiotic, despite their widely differing targets; however, these are highly phage-antibiotic and phage-host pairing specific. Screening across multiple clinical strains reveals that temperate phages can reduce the antibiotic minimum inhibitory concentration up to 32-fold, even in a resistant isolate, functionally re-sensitizing the bacterium to the antibiotic. Meropenem and tobramycin did not reduce the frequency of lysogens, suggesting a mechanism of action independent of the temperate nature of the phages. By contrast, ciprofloxacin and piperacillin were able to reduce the frequency of lysogeny, the former by inducing phages—as previously reported in *E. coli*. Curiously, synergy with piperacillin reduced lysogen survivors, but not by inducing the phages, suggesting an alternative mechanism for biasing the phage lysis-lysogeny equilibrium. Overall, our findings indicate that temperate phages can act as adjuvants in clinically relevant pathogens, even in the presence of antibiotic resistance, thereby drastically expanding their therapeutic potential.

**IMPORTANCE:**

The recent discovery that otherwise therapeutically unusable temperate phages can potentiate the activity of antibiotics, resulting in a potent synergy, has only been tested in *E. coli*, and with a single model phage. Here, working with clinical isolates of *Pseudomonas* and phages from these isolates, we highlight the broad applicability of this synergy—across a variety of mechanisms but also highlight the limitations of predicting the phage, host, and antibiotic combinations that will synergize.

## INTRODUCTION

The widespread use of antibiotics has selected for resistance, resulting in a decline in their effectiveness and a rise in untreatable infections (1). As a result, there has been a renewed interest in treatments with alternative modes of action such as bacteriophage (phage) therapy. Phages are bacterial-specific viruses (2) that hijack the host cell machinery and redirect it to synthesize phage components, resulting in host cell lysis and release of new infectious phage progeny (3). These can also synergize with multiple classes of antibiotics, known as “Phage-Antibiotic Synergy” (PAS), which is associated with changes in phage replication and an increase in phage production (4–7).

Most phage therapy work to date has been with “virulent” (strictly lytic) phages. In contrast with these, temperate phages can undergo an additional replication cycle known as lysogeny, a dormant state involving the integration of the phage genome into the bacterial host and replication along with it (8). The integrated phage is referred to as a prophage and a bacterium carrying a prophage is a lysogen. In addition to choosing between lysis and lysogeny at the time of infection, prophages are capable of exiting lysogeny and switching to lytic replication in response to external stressors in a process known as induction (8). This decision between lysis and integration, at the initial time of infection or later during dormancy, is facilitated by phage-encoded proteins in many well-studied temperate phage models (e.g., cI repressor in *Escherichia coli* phage Lambda) (9, 10). Even though this switch is genetically encoded, much of this decision is responsive to environmental factors (11–15). Of these, one of the most well known are antibiotics that trigger the bacterial SOS response (e.g., mitomycin C, fluoroquinolones, some beta-lactams) and result in phage induction through subsequent cleavage of the phage repressor protein (16–22).

Temperate phages have been overlooked for use in phage therapy because they present concerns of overgrowth of phage-resistant lysogens because of immunity; superinfection immunity (23, 24), mediated by the phage repressor protein, or superinfection exclusion, mediated by phage proteins that block DNA-entry (25). However, due to the narrow host range of many phages, in instances when lytic phages are difficult to find (26, 27) studies have had to employ virulent variants of temperate phages obtained through genetic engineering for the treatment of bacterial infections (28, 29). Interestingly, up to 75% of bacteria already contain a prophage in their genome (30, 31), greatly facilitating their discovery. The isolation and use of virulent mutants of temperate phages that can infect a lysogenic host have also been proposed for *P. aeruginosa* infections (32). These can also be of particular interest for pathogens such as *Clostridioides difficle* where strictly lytic phages have not been identified to date (26).

Studies examining the potential of temperate phages in therapy have been few and far between. Temperate phages of *Burkholderia cepacia* complex show synergistic interaction with other phages where their therapeutic potential inversely correlates with the frequency of lysogeny (33). *Burkholderia* temperate phage KS14 also synergizes with several antibiotic classes, as measured by an increase in plaque size and phage titer (6). In addition, temperate phages isolated from clinical strains of *Pseudomonas aeruginosa* were shown to decrease twitching motility, important for virulence, in lysogens (34). Administration of a temperate phage was able to reduce bacterial load and prevent toxin production in an *in vitro* human colon model of *C. difficile* infection (35). However, the study also reported increased spore formation with potential for increased risk of re-emerging infection. Several studies have also proposed the use of temperate phage cocktails for *C. difficile* (36, 37). In a hamster model, Nale et al. (37) reported that the phage cocktail not only reduced the colonization load of *C. difficile* but could also delay symptoms by 33 h (37).

While temperate phages are currently not ideal for monotherapy, the use of adjuvants that can bias their decision away from lysogeny and toward lytic replication can be promising for compassionate last-resort cases. A four-temperate phage cocktail combined with Ca2+ or Zn2+ reduced methicillin-resistant *S. aureus* load by 2.64-fold compared to the phage cocktail alone in a mouse model; however, the frequency of lysogeny was not reported (38). Knezevic et al. (39) demonstrated that temperate phage σ−1 combined with ¼ minimum inhibitory concentration (MIC) ceftriaxone reduced *P. aeruginosa* counts by ≥2 logs (39). The same effect was not observed with ciprofloxacin; a fluoroquinolone, gentamicin; a protein synthesis inhibitor, and polymyxin B; an outer membrane-targeting antibiotic. The study briefly noted a potential involvement of antibiotic-mediated phage induction.

Supported by the idea that SOS-response inducing antibiotics can act as phage inducers, we previously demonstrated that the combination of temperate phage HK97 and sublethal ciprofloxacin can synergistically reduce *E. coli* survivor count up to 10^8^-fold after an 18-h treatment, largely by inducing any lysogens that formed. This was dubbed temperate phage antibiotic synergy (tPAS) (40) and was generalizable to other antibiotics, including quinolones, anti-folates, and mitomycin C—all known to induce the bacterial SOS response (41). Interestingly, protein-synthesis inhibitors of several classes also show comparable synergy in this model, although these were determined to act by biasing the phage away from lysogeny during the initial infection, rather than by inducing lysogens (41).

With the reported efficacy of tPAS in *E. coli* demonstrating a substantial reduction in lysogeny, the major concern in the therapeutic use of temperate phages, here we systematically investigate its effectiveness across phages, hosts, and antibiotics in the clinically relevant pathogen *P. aeruginosa*.

## RESULTS AND DISCUSSION

### Temperate phages that synergize with ciprofloxacin are readily isolated from clinical strains

**Fig 1:**
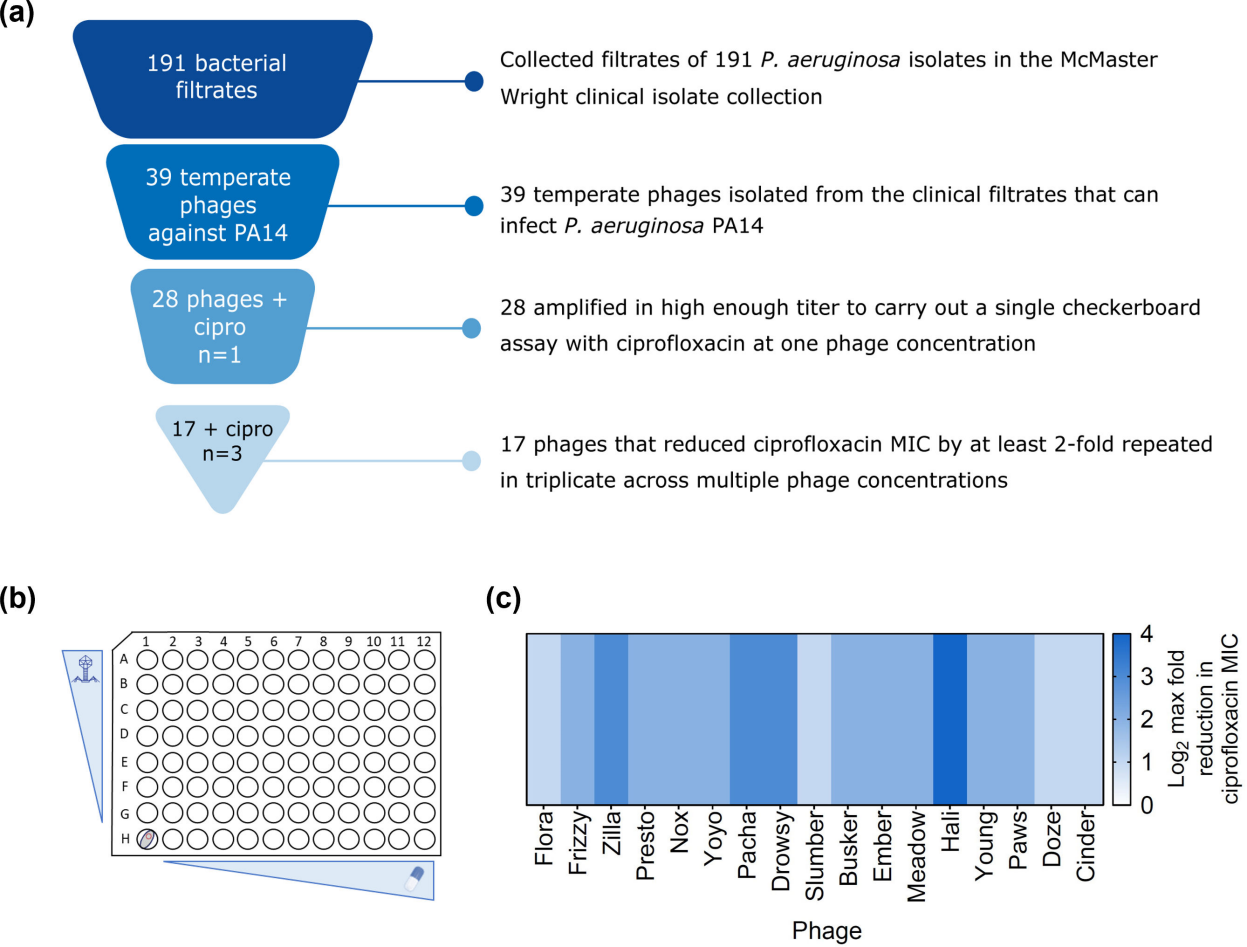
Temperate phages that synergize with ciprofloxacin can readily be isolated from clinical strains. (**a**) Workflow of isolation of temperate phages infecting *P. aeruginosa* PA14 from clinical strain collection and screening for synergy with ciprofloxacin in PA14. (**b**) Illustrative representation of checkerboard assay screening for synergy between a temperate phage and antibiotic. (**c**) Log_2_ maximum reduction in ciprofloxacin MIC achieved with the addition of a panel of temperate phages relative to no phage added control against *P. aeruginosa* PA14, plotted as a heat map (*n* = 3 biological replicate for all except *n* = 5 for phage Flora). The multiplicity of infection (MOI) range of 1.25–40 was tested in two-fold increments. The data represented are the maximum MIC reduction regardless of phage dose.

To establish tPAS in *P. aeruginosa*, we isolated 39 temperate phages that could infect the strain *P. aeruginosa* PA14 from filtrates of overnight cultures of 191 *P*. *aeruginosa* clinical isolates, highlighting the abundance and ease of isolation of these phages (Fig. 1a). This is consistent with many reports of isolation of temperate phages from clinical strains of *P. aeruginosa* and *C. difficle*, often aided by mitomycin C (42–44).

Of the 39 phages, 28 were amplified on *P. aeruginosa* PA14 to a high enough titer to screen for synergy with ciprofloxacin using a checkerboard assay in a single replicate (Fig. 1a and b). In all, 17 phages that reduced the antibiotic MIC by at least two-fold (limit of detection) were then repeated in biological triplicates. A summary of the screening with ciprofloxacin is shown in Fig. 1c as maximum fold reduction in the ciprofloxacin MIC achieved with the addition of the phage, regardless of the phage dose. All 17 phages reduced the ciprofloxacin MIC by at least two-fold, with the highest (16-fold) reduction achieved with phage Hali. These findings highlight that synergy between temperate phage and antibiotics can be achieved in *P. aeruginosa* with multiple phages, at least with ciprofloxacin, a known bacterial DNA-damaging phage inducer.

**Fig 2:**
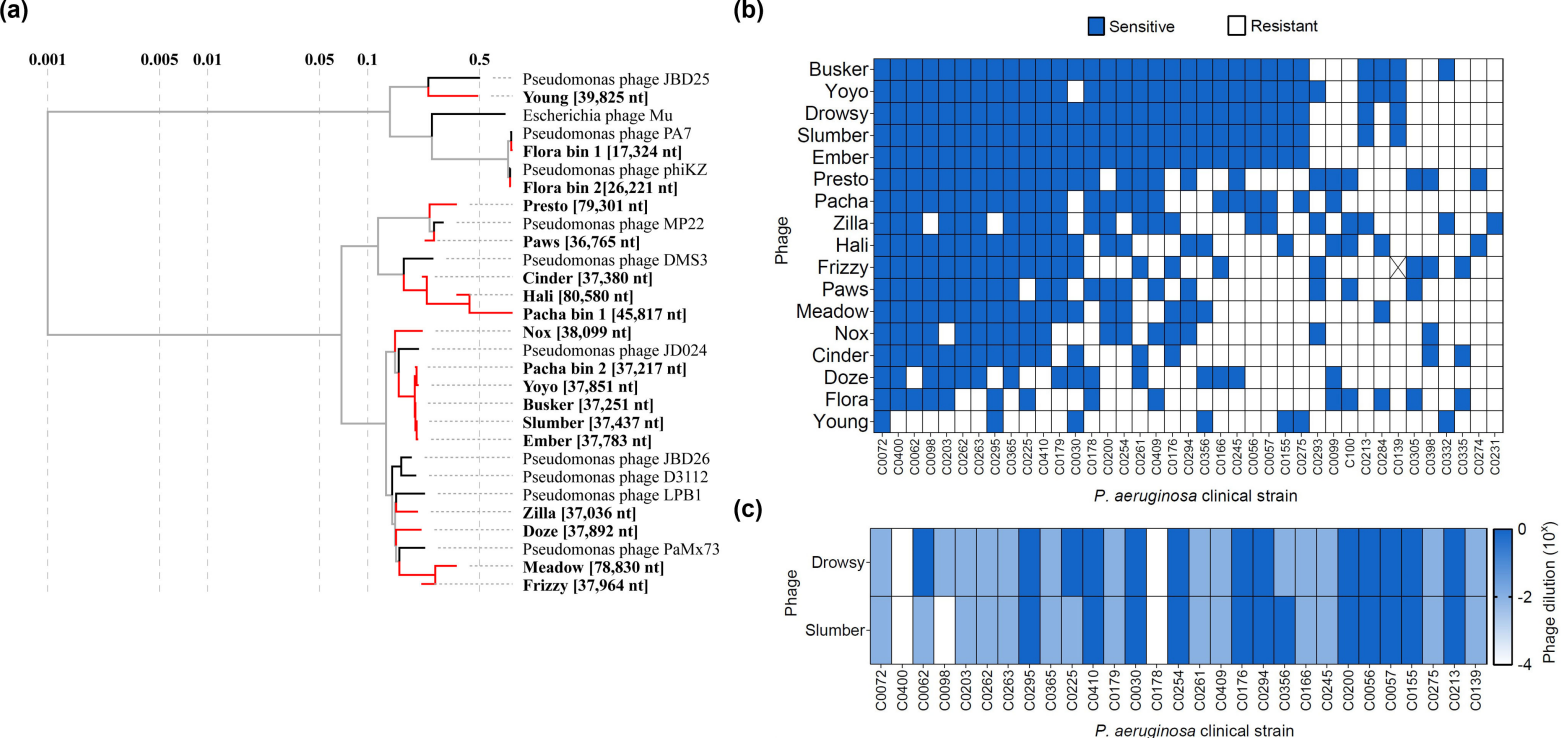
Newly isolated PA14 temperate phages are unique. (**a**) Phylogenetic tree of the isolated PA14 temperate phages. The tree was generated using ViPTree with a subset of related genomes. Branch lengths are indicated on a log scale. (**b**) Host range of the newly isolated PA14 temperate phages tested against 96 *P*. *aeruginosa* clinical strains. Each row represents a phage, ordered in largest to smallest host range, and columns represent a single *P. aeruginosa* isolate, ordered based on phage susceptibility. Blue denotes phage susceptibility. (**c**) Host range comparison of phage Drowsy and phage Slumber. The heat map depicts the 10-fold phage dilution on which plaquing was observed on a specific host, represented by the columns.

The 17 phages that exhibited a synergistic interaction with ciprofloxacin were further characterized using whole-genome sequencing and bacterial host range analysis. Recognizable phage genomes were obtained for all phages except phage Drowsy. Genome annotation predicted a transposase in 15 out of the 16 phages (not present in phage Flora) and these phages cluster closely with other *Pseudomonas* transposable temperate phages (Fig. 2a). Host range analysis carried out using 96 *P*. *aeruginosa* clinical isolates reveal that these phages exhibit broad host range, with phage Busker able to infect 31 out of the 96 strains tested (Fig. 2b). While phage Drowsy and phage Slumber exhibited a similar host range, the efficiency of plaquing for 4 out of 29 strains differed (Fig. 2c). Despite variations in host ranges—some of which can presumably be attributed to host-controlled variation, based on sequence similarity and phylogenetic analysis of phages Yoyo, Pacha, Slumber, Busker and Ember, phage Ember was kept as a representative of that cluster.

### tPAS with ciprofloxacin results in bacterial eradication through induction

**Fig 3:**
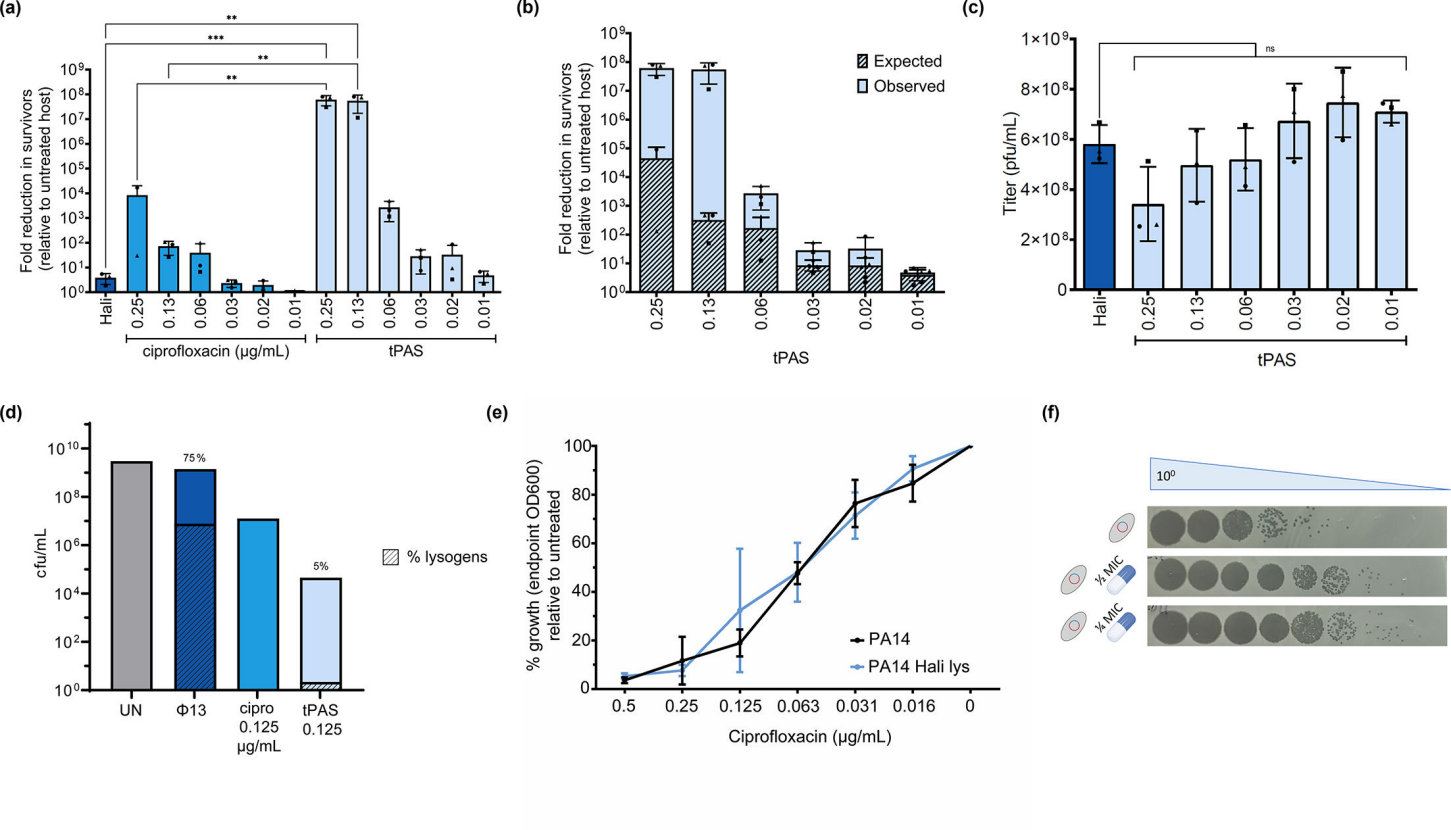
Phage Hali and ciprofloxacin synergize to eradicate *Pseudomonas* through induction. (**a**) Fold reduction in PA14 survivors after phage Hali [multiplicity of infection (MOI) of 15] and ciprofloxacin treatment relative to untreated host (mean ± SD). In the case that no survivors were detected, the data were set to the limit of detection (one colony). Each point indicates a biological replicate, denoted by the different shapes, counted in technical triplicates. Values were compared by a one-way ANOVA and Tukey post hoc test, with ^***^P-value 0.0001–0.001 and ^**^P-value 0.001–0.01. (**b**) Observed fold reduction in survivors (solid) versus the expected (diagonal line) effect. Expected effect was calculated by multiplying the phage alone reduction with antibiotic reduction for the corresponding antibiotic concentration. (**c**) Phage quantification from the overnight challenges (PFU/mL, mean ± SD). Each point indicates a biological replicate, denoted by the different shapes, counted in technical triplicates. All challenges were compared to phage alone using one-way ANOVA and Bonferroni’s test, with “ns” denoting not significant. (**d**) Survivor quantification (cfu/mL) of untreated PA14, challenged with phage Hali [multiplicity of infection (MOI) of 15] ± 0.125 ug/mL ciprofloxacin, where the strongest synergy was observed in a repeat experiment. Striped bars indicate the percentage of lysogens as determined using a lysogen stamp test, values also indicated above the bar. Twenty survivors of phage ± ciprofloxacin were streaked purified for lysogen testing. The height of the % lysogen bar is proportional to the height of the total cfu/mL bar. (**e**) Ciprofloxacin MIC curve of PA14 and PA14 phage Hali lysogen represented as percent growth (endpoint OD_600_) relative to untreated host, plotted as mean ± SD (*n* = 3 biological replicates, each in technical duplicates, except no antibiotic which was technical triplicates). (**f**) Representative phage quantification of PA14 phage Hali lysogen challenged with ½ and ¼ MIC ciprofloxacin.

While checkerboard assays do establish a clear synergistic effect, revealed as a reduction of the antibiotic MIC, they do not provide enough resolution to quantify this effect and determine the mechanism of bacterial growth inhibition. To better quantify this synergy over a range of antibiotic concentrations, we challenged *P. aeruginosa* PA14 with phage Hali and ciprofloxacin in liquid media. Survivors present after an 18 h challenge were plated with no selection for another 18 h. Fold reduction in survivor count relative to the untreated host was calculated for each challenge. There was a less than five-fold reduction in survivor count when challenged with phage Hali alone and a more pronounced dose-dependent ~10^4^-fold reduction with antibiotic at the highest concentration (Fig. 3a). These results highlight the poor efficacy of the phage and antibiotic alone in eradicating PA14.

By contrast, PA14 challenged with phage Hali in the presence of ciprofloxacin at the two highest concentrations resulted in approximately a 10^8^-fold reduction in survivors, corresponding to complete eradication. The number of survivors then decreased in a dose-dependent manner as the antibiotic concentration decreased. However, the clearest evidence for a synergistic effect is obtained by comparing the observed data to the expected multiplication of the independent effects of phage and antibiotic (Fig. 3b). Phage Hali and ciprofloxacin resulted in the strongest synergistic effect at sublethal 0.13 µg/mL with an observed reduction that is roughly 10^5^-fold higher than the expected effect. This effect is much higher than that observed with temperate phage σ−1 and ceftriaxone in *P. aeruginosa* ATTC 9027 (39). However, it agrees with our previous work performed in *E. coli* with model temperate phage HK97 and ciprofloxacin (40), although across a smaller range of antibiotic concentrations.

To elucidate the mechanism through which phage Hali-ciprofloxacin pairing results in bacterial killing, we quantified phages in the filtrates of the phage-alone and tPAS challenges from the overnight survivor quantification assay (Fig. 3c). We observed no significant difference in phage titer in the presence of the antibiotic compared to the phage alone challenge suggesting that phage Hali-ciprofloxacin synergy is not a result of a substantial increase in phage replication.

To determine whether tPAS was instead biasing the phage lysis-lysogeny decision, we repeated the assay across a shorter range of antibiotic concentrations, purifying survivors from phage alone and a single antibiotic concentration where we observed the strongest synergy with regard to reduction in survivor count (Fig. 3d). Phage alone resulted in 75% lysogens, which reduced to 5% in the presence of ciprofloxacin. The interaction works at the level of biasing the phage lysis-lysogeny equilibrium, potentially at the level of induction since antibiotics are well known to result in induction.

To confirm whether PA14 lysogen of phage Hali can be induced with sublethal ciprofloxacin, we challenged wild type and lysogen with ciprofloxacin and quantified phages in the filtrates. Unlike in prior *E. coli* phage HK97 work (40), the PA14 phage Hali lysogen is not more sensitive to ciprofloxacin than the wild type (Fig. 3e), despite the roughly 100-fold increase in phage titer, relative to baseline spontaneous induction, in filtrates of lysogen grown with sublethal ciprofloxacin (Fig. 3f). The disagreement between this and lack of increase in phage titer observed in overnight challenges could be explained by previous reports that the inherent frequency of lysogeny is typically very low. For example, for Lambda in nutrient broth at 37°C it is typically <1% (45), but it is the regrowth of these lysogens that result in large resistant populations. This means that even by forcing all the phage to adopt a lytic cycle, the phage titer would only be expected to increase by almost 1%, which is well below our limit of detection.

Overall, our results indicate phage Hali and ciprofloxacin synergy results in bacterial killing by preventing the expansion of lysogen colonies likely through induction, where lysogens form but are subsequently induced in the presence of sublethal antibiotic, like that reported in *E. coli* with phage HK97 and ciprofloxacin (40).

### tPAS can be achieved even with delayed antibiotic administration

**Fig 4:**
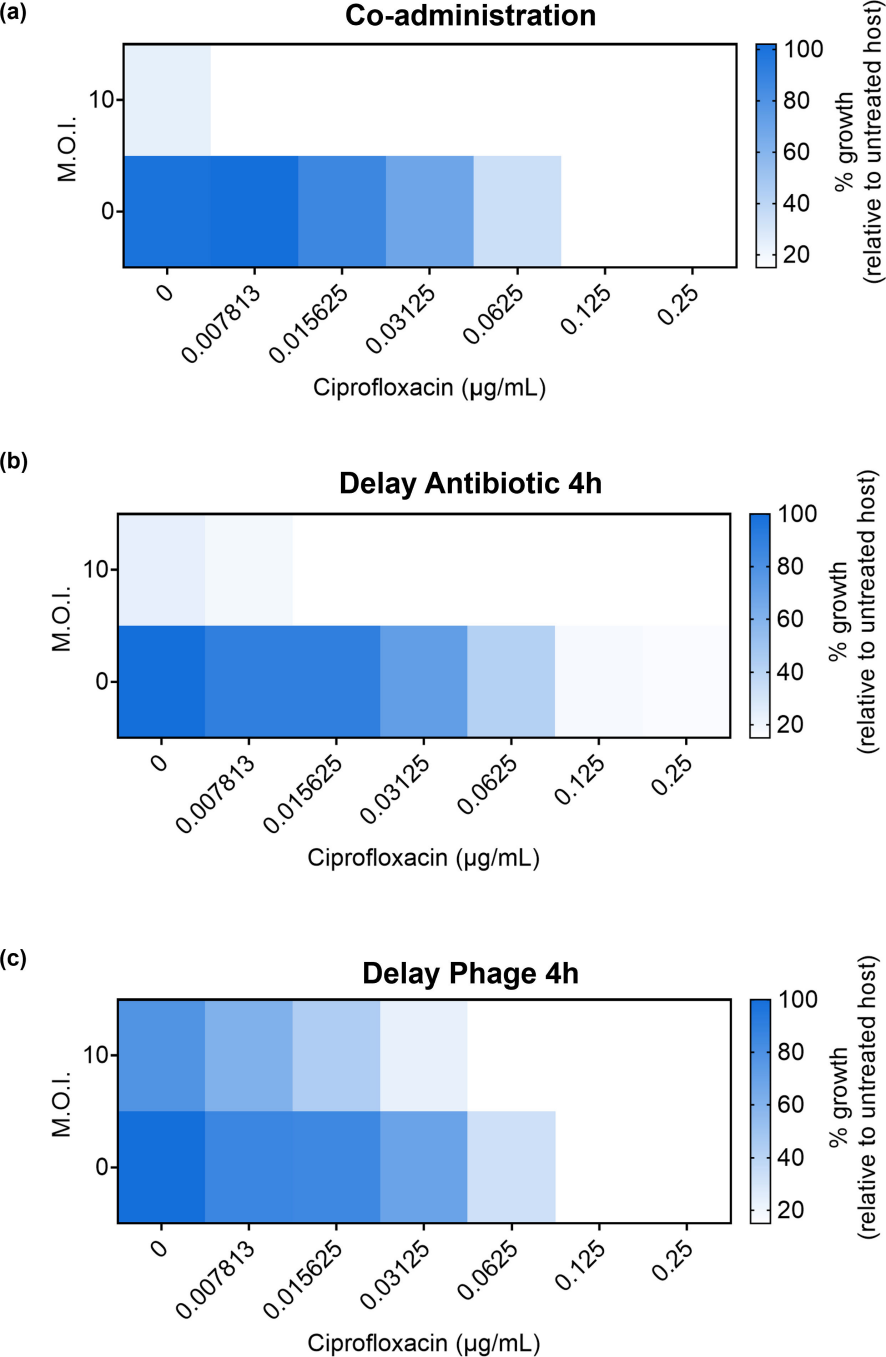
Temperate PAS can be achieved by delaying the antibiotic. Checkerboard assay of phage Hali [multiplicity of infection (MOI) of 10] and ciprofloxacin in PA14 with (**a**) simultaneous, (**b**) delayed antibiotic by 4 h, or (**c**) delayed phage by 4 h. Endpoint growth (OD_600_) relative to untreated host, plotted as a heatmap (*n* = 3 biological replicates, each in technical triplicates).

With a clear synergistic interaction observed with the co-administration of phage Hali and ciprofloxacin, we sought to investigate whether both agents need to be simultaneously present to achieve synergy. To first test whether the antibiotic needs to be present when the phage infects, we monitored bacterial growth after simultaneous phage and antibiotic administration (Fig. 4a) or delayed antibiotic treatment by 4 h where the antibiotic likely only has lysogens to interact with (Fig. 4b). In these assays, we report the functional change in the MIC of the antibiotic in the presence of the phage but do not claim that the phage is affecting the antibiotic—the relative increase (or decrease) in the potency of the antibiotic is almost certainly a result of the interaction of the antibiotic with the phage and not the other way around.

Delaying the antibiotic by 4 h results in an increase in antibiotic MIC compared to administering it alone at time zero (Fig. 4b). However, a comparable level of synergistic reduction in ciprofloxacin MIC was still observed in the presence of phage Hali when the antibiotic was delayed. Ciprofloxacin does not necessarily need to be present when the phage infects to achieve the same levels of tPAS as co-administration. Taken together with the frequency of lysogeny (Fig. 3d), the results highlight that phage Hali and ciprofloxacin synergy works through biasing lysis-lysogeny decision at the level of induction.

We also tested the effects of delaying the phage to investigate whether pre-stressing the bacteria with antibiotics prior to phage treatment would result in synergy. Delaying the phage by 4-h results in only a two-fold reduction in ciprofloxacin MIC (Fig. 4c). While the antibiotic does not necessarily need to be present at the time of phage infection, the opposite is not true. By delaying the phage, we drastically reduce the range of antibiotic concentration at which tPAS is observed.

It is important to note that the efficacy of delayed administration is likely dependent on the phage-antibiotic pairing. *P. aeruginosa* virulent phage JG024 demonstrates synergy with ciprofloxacin even with a 1 h delay of either agent (46). The effect is lost if the treatment occurs after 6 h. By contrast, with ceftriaxone, only delaying phage JG024 by 1 h can inhibit bacterial growth and delayed antibiotic has no effect. Delaying ciprofloxacin (8× MIC) and gentamicin (1× MIC and 8× MIC) 6 h post-lytic phage treatment also reduced viable cell count >10^2^ cfu/mL (limit of detection), better than sequential treatment, in 48 h mono species *P. aeruginosa* biofilm (47). With regards to tPAS, the extent to which one of the agents can be delayed would presumably depend on the rate at which lysogens form in a specific host.

### tPAS is generalizable across phage-antibiotic pairings

**Fig 5:**
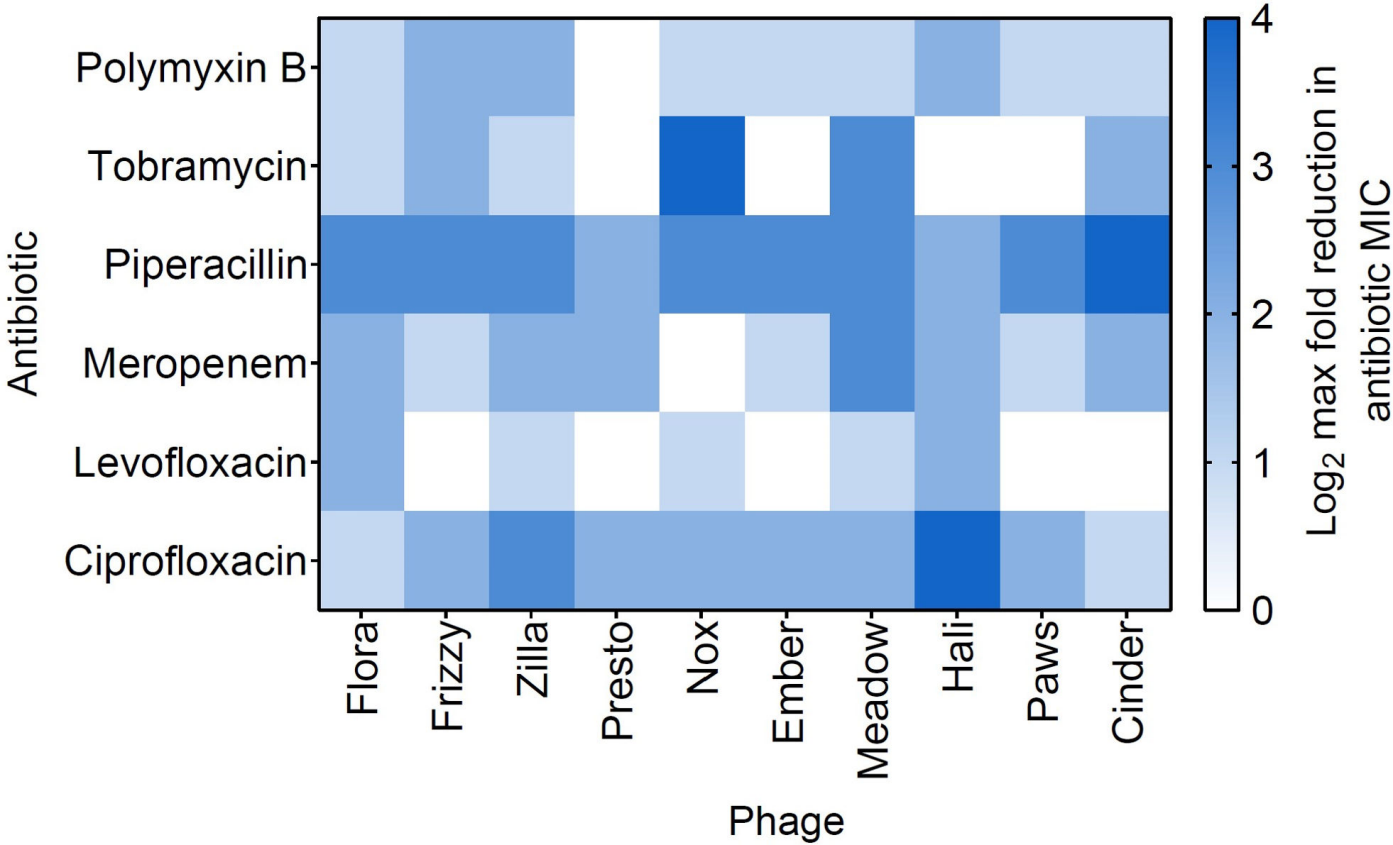
PAS is generalizable to other temperate phage-antibiotic pairings. Ten PA14 temperate phages were screened with two fluoroquinolones (ciprofloxacin and levofloxacin), two beta-lactams (meropenem and piperacillin), an aminoglycoside (tobramycin), and polymyxin B. Ciprofloxacin data are reproduced from Fig. 1c as a reference. Log_2_ maximum reduction in antibiotic MIC achieved in the presence of PA14 temperate phages relative to no phage control, plotted as a heat map (*n* = 3 biological replicates, with a few *n* = 4 exceptions). Data represented are the maximum MIC reduction regardless of phage dose [multiplicity of infection (MOI) range of 1.25–40 was tested in two-fold increments].

Having established this interaction with ciprofloxacin, we sought to test the generalizability across antibiotics, covering three more antibiotic classes in addition to fluroquinolones (Fig. 5). These antibiotic classes were selected for their reported ability to either induce phages or their clinical relevance as anti-pseudomonal drugs. Beta-lactams are cell wall synthesis inhibitors that bind to penicillin-binding protein (PBP) to prevent crosslinking (48). While these have been shown to result in phage induction in multiple bacterial hosts (21, 49), the exact mechanism through which they induce phages, directly or indirectly through the bacterial SOS response (50–52), remains unclear. The beta-lactams, meropenem and piperacillin, are commonly used in the clinic to treat *Pseudomonas* infection, where the latter is combined with a beta-lactamase inhibitor, tazobactam (53).

In addition, aminoglycosides inhibit protein synthesis by binding to the A site of 16S ribosomal RNA, inhibiting translocation and resulting in protein mistranslation (54). Since phages need host protein machinery for replication, protein synthesis inhibitors are not expected to synergize with antibiotics. Kanamycin antagonizes replication of *E. coli* phage T3, demonstrated as a decrease in efficiency of plaquing, bacterial growth, and biofilm biomass, by decreasing phage burst size independent of changes in phage adsorption (55). Similarly, both kanamycin and apramycin reduced the efficiency of plaquing of temperate phage Lambda by up to 1,000-fold (56). Selected protein synthesis inhibitors were also reported to antagonize *P. aeruginosa, S. aureus, and Enterococcus faecium* phages (57). Contrary to these studies, gentamicin and kanamycin both synergize with phage HK97 in *E. coli* by biasing the initial phage decision toward lysis at the time of infection (41). To investigate whether a similar synergy between aminoglycoside and temperate phages can be observed in *P. aeruginosa,* we performed our assays with tobramycin, frequently used as an inhaled treatment for cystic fibrosis patients (58). Our screen also includes polymyxin B, an older outer membrane-disrupting antibiotic which works through binding to lipopolysaccharide (59), which has regained interest as one of the last resort drugs for severe infections.

We observed synergy with all antibiotics, despite their vastly different bacterial targets, with most of the phages working particularly well in combination with piperacillin (Fig. 5). While some of these antibiotics have not been previously reported to interact specifically with temperate phages, a strong synergy between virulent phages and several cell wall synthesis inhibitors (16/25 antibiotics tested), including piperacillin and meropenem, and protein synthesis inhibitors (5/25 antibiotics tested), including tobramycin, has been previously reported in *P. aeruginosa* (60, 61). Polymyxin B has also been reported to work in combination with virulent phages for treating *S. aureus* (62). However, we did not observe a consistent synergistic pattern with antibiotics of the same drug class, indicating that PAS here is phage-antibiotic pairing specific, previously also reported with a lytic phage and several antibiotic classes in extraintestinal pathogenic *E. coli* (63). Nonetheless, this drastically expands the library of compounds that can be used in combination with temperate phages for therapeutic use, even with antibiotics not previously known to induce phages.

### Synergy with piperacillin reduces the frequency of lysogeny, but not through induction

**Fig 6:**
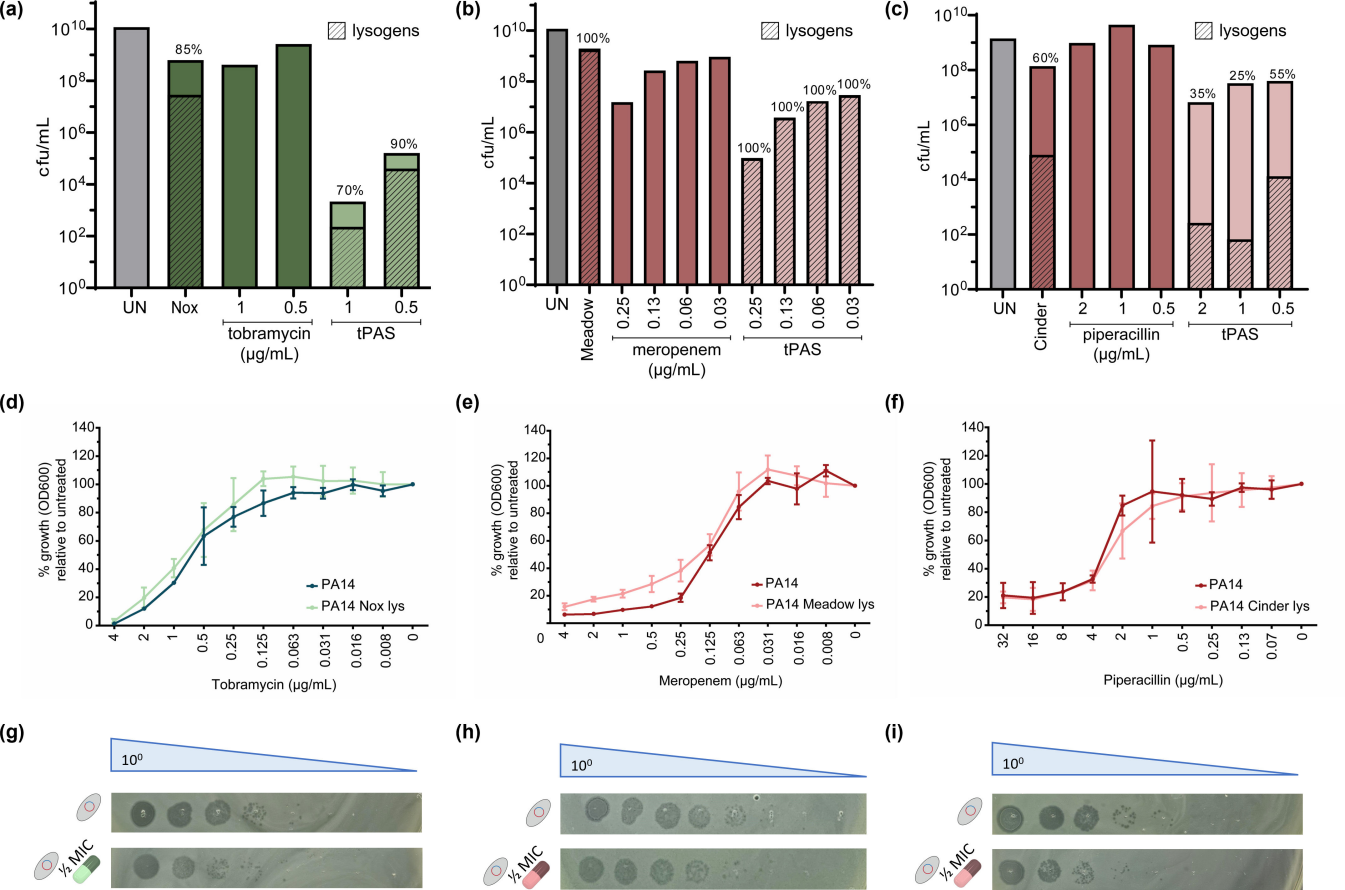
Piperacillin reduces the frequency of lysogeny but not through induction. (**a–c**) Survivor quantification (cfu/mL) of PA14 challenged with individual and combination (**a**) phage Nox [multiplicity of infection (MOI) 2.5] + tobramycin, (**b**) phage Meadow [multiplicity of infection (MOI) 15] + meropenem, and (**c**) phage Cinder [multiplicity of infection (MOI) 15] + piperacillin. Striped bars represent the percentage of lysogens as determined using a lysogen stamp test, values also indicated above the bar. Twenty survivors of phage ± antibiotic were streaked purified for lysogen testing. The height of the % lysogen bar is proportional to the height of the total cfu/mL bar (*n* = 1 biological replicate). (**d–f**) Antibiotic sensitivity of wild type PA14 and (**d**) phage Nox lysogen with tobramycin, (**e**) phage Meadow lysogen with meropenem, and (**f**) phage Cinder lysogen with piperacillin. MIC is represented as percent growth (endpoint OD_600_) relative to untreated host, plotted as mean ± SD (*n* = 3 biological replicates for tobramycin and piperacillin, *n* = 4 for meropenem, each in technical triplicates). (**g–i**) Representative phage quantification ± ½ MIC antibiotic of PA14 (**g**) phage Nox lysogen with tobramycin, (**h**) phage Meadow lysogen with meropenem, and (**i**) phage Cinder lysogen with piperacillin (*n* = 3 biological replicates, each with single technical replicate).

With a clear synergy observed with several antibiotics, we looked to confirm whether the interaction with these other classes of antibiotics also operates at the level of biasing the phage lysis-lysogeny equilibrium. To test this, we purified survivors from our overnight survivor quantification assay performed for three other phage-antibiotic pairings. Phage Nox combined with tobramycin was able to reduce survivor counts by 3–4 logs; however, there was only a 15% reduction in lysogeny observed at the highest concentration tested (Fig. 6a). Meropenem combined with phage Meadow synergistically reduced survivor count but did not reduce the frequency of lysogeny (Fig. 6b). In comparison, while there was not an impressive reduction observed in survivors for phage Cinder-piperacillin, there was a clear reduction in lysogeny from 60% for phage alone down to 25% in the presence of sublethal piperacillin (Fig. 6c). The contrasting results of meropenem and piperacillin, belonging to the same drug class, further support that tPAS is phage-antibiotic pairing specific.

To confirm whether the observed synergy and reduction in lysogeny could be a result of the ability of the antibiotic to result in induction, we tested the sensitivity of the wild type and the lysogen for their respective antibiotic (Fig. 6d through i). None of the lysogens were more sensitive to the antibiotic or released more phages at sublethal doses relative to spontaneous induction after 18 h. This explains the lack of reduction in lysogeny observed with meropenem or tobramycin, where we hypothesize the synergy observed is likely driven by the mechanism of traditional PAS instead. By contrast, synergy with piperacillin does operate by biasing the lysis-lysogeny decision; however, it is not at the level of induction. Piperacillin potentially works at the level of biasing the decision at the initial time of infection, like that reported with *E. coli* phage HK97 and gentamicin (41). The observed effect with piperacillin could also be attributed to its selective affinity for PBP3, unlike meropenem which preferentially binds PBP4 (64), and/or its ability to result in cell filamentation (65–67), where increased cell volume shows a decrease in the probability of lysogeny in *E. coli* phage Lambda (68). This is the first report that we know of piperacillin’s ability to influence the phage lysis-lysogeny balance. This provides further proof that tPAS can be achieved with non-phage-inducing antibiotics.

### tPAS is generalizable across clinical strains, even antibiotic-resistant ones

**Fig 7:**
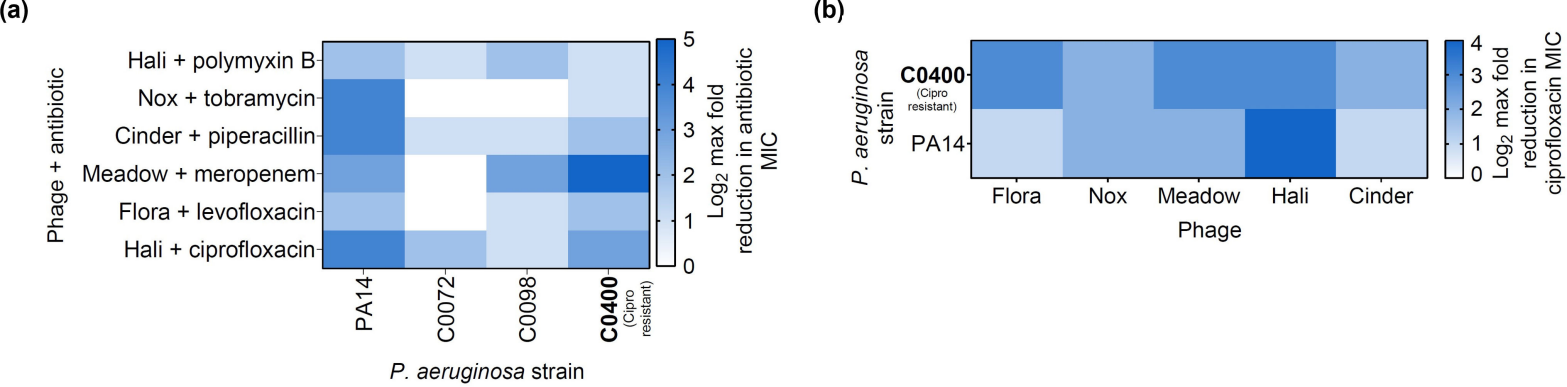
PAS with temperate phages works in clinical isolates. (**a**) Six of the strongest pairings from PA14 screen (phage Hali + ciprofloxacin, phage Flora + levofloxacin, phage Meadow + meropenem, phage Cinder + piperacillin, phage Nox + tobramycin, phage Hali + polymyxin B) were tested for synergy in *P. aeruginosa* clinical strains based on host range in Fig. 2b. Result reported as log_2_ reduction in antibiotic MIC calculated from checkerboard assay (*n* = 3 biological replicates). (**b**) Log_2_ reduction in ciprofloxacin-resistant strain C0400 MIC with the addition of five temperate phages, regardless of phage dose [multiplicity of infection (MOI) range of 1.25–40 tested in two-fold increments]. Average maximum reduction (*n* = 3 biological replicates) in MIC shown as heat map as determined from checkerboard assays. Isolate in bold is classified as antibiotic-resistant strains in the clinical database using standard laboratory reporting. (**a and b**) PA14 screen data from Fig. 1c and (**b**) C0400 phage Hali data from panel a are reproduced as reference.

To establish whether the synergy is generalizable across hosts, we identified the six strongest phage-antibiotic pairings, one for each antibiotic in Fig. 5, and tested for synergy across multiple clinical strains, including both antibiotic-sensitive and -resistant ones. Figure 7a shows the maximum reduction in antibiotic MIC achieved with the addition of the phage across three isolates that were sensitive to all phages used. We also carried this out with five other clinical strains that were sensitive only to some overlapping phages (Fig. S1).

Our findings show that the phage-antibiotic combinations that were initially identified to synergize in PA14 expand across multiple clinical isolates of *P. aeruginosa*, even in the multi-drug-resistant strain C0400. The reported ciprofloxacin resistance of this strain is consistent with our data, as the strain showed four-fold higher MIC than PA14 (Fig. S2a). We observed a reduction in ciprofloxacin MIC of this isolate even when combined with four other phages at levels much higher than those observed in the initial PA14 screen (Fig. 7b). The co-administration of temperate phage results in as high as a 16-fold reduction in MIC, bringing it down to levels comparable to PA14, re-sensitizing the isolate to the antibiotic. This is in line with previous observations that PAS can be achieved independent of the antibiotic-resistant nature of the bacteria (6, 61, 63, 69, 70).

Strain C0400 is predicted by the Comprehensive Antibiotic Resistance Database Resistance Gene Identifier software to be ciprofloxacin resistant due to the presence of a resistance-nodulation-cell division antibiotic efflux pump (71). Since many antibiotic targets also play a crucial role in phage replication and induction, the role of the exact mechanism of antibiotic resistance should be considered when investigating phage-antibiotic synergy. For example, efflux pumps can serve as phage receptors; hence, the development of phage resistance at the level of the surface receptor can result in re-sensitization to the antibiotic (72). This evolutionary tradeoff was used to successfully treat a 76-year-old male patient with *P. aeruginosa*-infected aortic graft using a combination of lytic phage OMKO1, which binds efflux pump, and ceftazidime.

However, the phages isolated in this study are temperate in nature where regrowth of lysogens presents a major concern as opposed to surface receptor mutants. Despite originally being resistant to ciprofloxacin, host C0400 Hali lysogen displays a higher antibiotic sensitivity, compared to wild type C0400 and PA14 (Fig. S2a) with significant increases in phage titer at sublethal ciprofloxacin (Fig. S2b). The increase in sensitivity of C0400 lysogen but lack of difference in PA14 lysogen ciprofloxacin sensitivity (Fig. 3e) could potentially be explained by host-specific factors. Since transposable phages are notorious for different integration sites, we also tested ciprofloxacin induction in independent lysogens, where our earlier findings suggest that it is the main phenotype driving tPAS with ciprofloxacin. We continued to detect ciprofloxacin-triggered induction in other isolated C0400 Hali lysogens (not shown). These findings further support the hypothesis that Hali-ciprofloxacin synergy prevents lysogen overgrowth through antibiotic-mediated induction, regardless of host. Our results highlight that tPAS can be achieved in clinical strains of *P. aeruginosa,* irrespective of their antibiotic-sensitivity profile.

### The frequency of lysogeny and antibiotic-mediated induction does not predict the ability of a temperate phage to synergize with an antibiotic

With proof that tPAS is working through biasing of the phage lysis-lysogeny equilibrium and a panel of phage-antibiotic pairings that showed efficacy in various hosts, we investigated whether there are factors that can predict tPAS. We hypothesized that the frequency with which a phage lysogenizes its host or its susceptibility to induction with the antibiotic would serve as predictors of tPAS.

To determine whether the ability of a phage to form stable lysogens in a specific host correlates with the level of synergy, measured as a reduction in MIC in the checkerboard assays, we infected host PA14 with seven phages and host C0400 with five phages in solid media and quantified frequency of lysogeny in the survivors of the phage challenge (Fig. S3a). The frequency of lysogeny varied in PA14 ranging from as low as 26% for phage Meadow to 100% for three other phages (phage Zilla, Drowsy, and Cinder). In comparison, all five phages exhibited 100% lysogeny in host C0400. However, there was no correlation between the frequency of lysogeny and the level of synergy observed with ciprofloxacin. It is important to note here that the frequency of lysogeny among survivors does not reflect only the actual frequency of such an event, but only the relative frequency within survivors. If the host cell has a low-cost alternative for resistance, we would find a low frequency of lysogeny among survivors, even if the actual rate at which lysogeny events occurred were quite high. The frequency of lysogeny here was also tested on solid media and therefore may differ if tested in broth.

In addition, we also investigated the ability of sublethal ciprofloxacin to induce these prophages (Fig. S3b). All tested lysogens of PA14 and C0400 were ciprofloxacin inducible, resulting on average in a 10–1,000-fold increase in phages released, except for the C0400 phage Flora lysogen. Similarly, when correlated with the level of synergy as measured by a decrease in MIC, the level of ciprofloxacin-mediated phage induction did not correlate with the magnitude of the synergy observed. This was only tested at one sublethal antibiotic concentration which may not be the optimal dose for induction with a given lysogen-antibiotic pairing. Induction was also measured at the endpoint after 18 h, where the effect may have been entirely lost in some cases as a result of phage re-adsorption.

Our results show that neither the frequency of lysogens among survivors nor the extent to which the antibiotic results in induction correlate strongly with levels of synergy observed and therefore cannot predict if and how strongly a temperate phage will synergize with a particular antibiotic.

### Conclusion

Here we evaluate the generalizability of tPAS against *P. aeruginosa*. Temperate phages can be easily isolated from clinical strain collections and synergize with multiple clinically relevant antibiotics, irrespective of antibiotic target. This synergy can functionally lower antibiotic MICs up to 32-fold and can do so even in resistant strains, functionally sensitizing them to the antibiotic. Mechanistically, some of the observed synergies are not temperate phage specific, while others—notably ciprofloxacin, work at the level of induction in agreement with previous reports in a single phage in *E. coli* (40). Excitingly, piperacillin can also bias phage lysis-lysogeny equilibrium but appears to be doing so by biasing the initial lysis-lysogeny decision—in a manner akin to that reported for protein synthesis inhibitors for a single phage in *E. coli* (41). While temperate phages have been discarded in therapy due to their ability to lysogenize, the use of sublethal antibiotics can serve to bias away from lysogeny. This is both phage-host and phage-antibiotic pairing specific, and difficult to predict using factors inherent to the interaction between the two players. More work deciphering the mechanisms underlying these interactions would be necessary for any practical applications, to guide antibiotic and phage selection.

## MATERIALS AND METHODS

### Bacterial strains and growth conditions

*P. aeruginosa* strain PA14 was kindly gifted to us by Dr. Lori Burrows at McMaster University. Clinical strains of *P. aeruginosa* were obtained from the McMaster IIDR Wright clinical isolate collection. Bacterial strains were grown in 10 mL of lysogeny broth (LB) at 37°C with 130 rpm shaking (Ecotron, Infors HT, Quebec, Canada). For growth on solid media, 1% (wt/vol) of LB agar and 0.75% (wt/vol) of LB soft agar was used. All plates were incubated at 37°C from overnight growth.

For each experiment, same-day culture was grown to an optical density (OD_600_) of 0.2 from a 1:100 dilution of an overnight culture. OD_600_ was measured using Thermo Fischer Scientific Spectronic 20D+ (Waltham, MA, USA).

### Phage isolation, propagation, and titration

In total, 191 *P. aeruginosa* clinical strains were grown overnight at 37°C in LB broth in a 96-well plate from frozen stocks. The plates were filtered using the Millipore MultiScreen_HTS_ vacuum manifold (Catalog MSVMHTS00, Darmstadt, Germany) with a Millipore Sigma MultiScreen_HTS_ High Volume 96-well 0.45-µm filter plate (Catalog MVHVN4525, Darmstadt, Germany). Approximately 2 µL of the undiluted filtrates was spotted on a 1% LB agar Nunc Omnitray single-well plate with a 15 mL 0.75% agar overlay containing 1 mL overnight culture of PA14. This assay would capture all antimicrobial components.

To identify phages, any filtrates that resulted in the clearing were then confirmed by spotting serial dilutions where a phage would dilute to a single plaque as opposed to other bactericidal entities. Briefly, an agar overlay of 300 µL of PA14 overnight culture into 3 mL of 0.75% molten agar was spread onto 1% LB agar petri plate. Ten-fold dilutions (10^0^–10^−7^) of the filtrates were prepared in 1× phage buffer and 3 µL was spotted on the overlay.

The phages were amplified in LB broth to increase the titer. Primary amplification was carried out using frozen stock of the bacteria and phage inoculated in 10 mL of LB broth and incubated for 18 h. Secondary amplification was carried out by challenging the same-day grown culture at OD_600_ 0.2 with 50 µL of the primary amplification. Phages were titrated on the respective host using a spot test and standard plaque assay.

To determine the multiplicity of infection (MOI), an OD_600_ vs colony-forming unit (cfu/mL) standard curve was carried out for each bacterial strain. The optical density of the same-day culture was measured at 30-min intervals and 50 µL of culture was sampled at every hour, diluted 10-fold in LB, and 100 µL was plated on a 1% LB plate using glass beads. MOI was calculated as $\frac{phagetiter\left(\frac{pfu}{mL}\right)\times phagevolume(mL)}{bacterialtiter\left(\frac{cfu}{mL}\right)\times volume(mL)}$.

For the clinical isolate screen, phages were amplified on the host being tested if the efficiency of plaquing was 2 log_10_ or lower relative to PA14. If not, lysate prepared on PA14 was used for the checkerboard screens.

### Minimum inhibitory concentration

The minimum inhibitory concentration of each antibiotic was determined using a modified microtiter assay. Ciprofloxacin (hydrochloride) was obtained from Cayman Chemicals (Catalog 14286–5, Ann Arbor, Michigan, USA), levofloxacin from Cedarlane (Catalog 20382-1, Burlington, ON, Canada), meropenem from Sigma-Aldrich (Catalog PHR1772, Oakville, ON, Canada), piperacillin from Sigma-Aldrich (Catalog PHR1805, Oakville, ON, Canada), tobramycin from Sigma-Aldrich (Catalog PHR1079, Oakville, ON, Canada), and polymyxin B sulfate from EMD Millipore (Catalog D46530, Oakville, ON, Canada). In a narrow 96-well plate (Corning, Product Number 3370, ME, USA), 100 µL of the same day culture was combined with antibiotic stock and LB broth in a final volume of 250 µL. The plate was incubated for 18 h at 37°C with no agitation. The endpoint OD_600_ was measured using the Epoch 2 microplate spectrophotometer (BioTek Instruments, Inc., VT, USA) with a 10-sec double orbital shake before reading. MIC was the lowest antibiotic concentration that resulted in no growth and was re-evaluated for every new batch of antibiotic stock prepared.

### Checkerboard assay

The same-day culture grown to OD 0.2 was challenged with two-fold dilutions of the phage on the vertical axis and two-fold dilution of the antibiotic on the horizontal axis in a narrow 96-well plate. MOI range tested for each phage was 40–1.25. The antibiotic concentrations tested changed depending on the MIC. For each checkerboard performed, the culture, phage, and antibiotic volume were fixed to 25 µL, 62.5 µL, and 12.5 µL, respectively, to achieve the desired MOI and antibiotic concentration. Untreated host and LB-only were used as growth controls. Plates were incubated for 18–20 h at 37°C. OD_600_ was used to measure growth in each plate using a BioTek Epoch 2 microplate spectrophotometer. The lowest antibiotic concentration that resulted in no growth (approximately 15% or less OD_600_ compared to the untreated control) was used as MIC. Checkerboard assay data were represented as the maximum reduction in antibiotic MIC that was achieved with the addition of the phage, regardless of MOI.

### Phage genome sequencing and analysis

Phage DNA extraction, DNA library preparation, sequencing, and analyses were carried out as described in reference (73). Briefly, 500 µL of phage lysate (≥10^8^ pfu/mL) was treated with DNase I, RNase, and DNase I reaction buffer and incubated at 37°C for 30 min followed by DNase and RNase inactivation at 65°C for 10 min. Next, proteinase K and 2% final volume SDS were added and incubated for 1 h at 37°C to denature protein and break open phage capsid. Following incubation, the mixture was split into two aliquots, and an equal volume of phenol-chloroform was added. The mixture was centrifuged for 10 min at >13,000 rpm, the supernatant was collected and treated with 1/10^th^ vol of ammonium acetate and one volume of −20°C isopropanol, followed by another round of 10-min centrifugation. The pellet was resuspended in −20°C 70% ethanol and centrifuged for 10 min. After decanting the supernatant, the new pellet was air-dried for 15 min and rehydrated in an elution buffer overnight at 4°C. The DNA concentration was measured using an Invitrogen Qubit 4 Fluorometer (ThermoFisher Scientific, Waltham, MA, USA).

DNA libraries were prepared for sequencing using the NEBNext Ultra II DNA Library Prep Kit (New England Biolabs, catalog no. E7645S, Massachusetts, USA) with a modified version of the Derakhshani et. al. (2020) protocol as outlined in previous reference (73). Sequencing was carried out using MiSeq with paired-end 2 × 300 reads at the McMaster Metagenomics Facility (Ontario, Canada). Genome assembly and analysis were carried out using the pipeline previously outlined (73). Briefly, quality assessment on raw reads was carried out using FastQC v0.11.8 (Andrews, 2010) before and after trimming with Trimmomatic v0.38 (Bolger et al.*,* 2014). The trimmed reads next go through a series of steps in which *de novo* assembly is performed using metaSPades v3.13.0 (Meleshko, 2017), and the sequence is predicted as phage or not. The last step in the pipeline involves analysis which includes within-sample comparison to determine how similar samples are to each other and genome annotation using RASTtk v1.3.0 (Brettin, 2019). The tree was generated using VipTree version 4.0 (https://www.genome.jp/viptree/) using the default setting after which a smaller subset of phages was selected to regenerate a smaller tree.

### Host range analysis

The phage host range was carried out as described previously (73). Briefly, a micro plaque assay was carried out using the Singer Rotor HDA. Ninety-eight clinical isolates of *P. aeruginosa* were inoculated from frozen in 1 mL LB in a deep 96-well plate and grown overnight. Forty-five microliters of each isolate was transferred into a 384-well plate such that each of the four quadrants of the 384-well plate were replicates. One 384-well plate was prepared for each phage where each quadrant contains 45 µL of either undiluted, 10^−2^, 10^−4^, and 10^−6^ phage lysate dilution prepared in LB broth. Stamping plates were prepared with 25 mL of 1.5% LB agar per plate. Using the Singer Rotor HDA, culture was stamped first, followed by phage directly on top at a 1,536 density (four replicates per phage dilution-host pairing). One plate with only culture stamped was used as a control. The plates were wrapped in plastic bags and incubated for 18 h at 37°C. Phage sensitivity and efficiency of plaquing, the lowest dilution at which plaques were observed, were noted for each phage-host pairing.

### Survivor quantification assay

Overnight challenge survivor quantification assay was performed as previously described (40). Briefly, same-day culture of PA14 was challenged with phage (MOI of 15 for phage Hali, Meadow, and Cinder, and MOI of 2.5 for phage Nox) and/or two-fold dilutions of antibiotic in a final volume of 1 mL. The untreated host was used as a growth control. Challenges were incubated at 37°C with 130 rpm shaking for 18 h, following which 100 µL of 10-fold dilutions carried out with LB broth was added to 5 mL of 0.75% LB and spread onto an empty Petri plate. Survivors were counted after a overnight incubation to determine the fold reduction in survivor count relative to the untreated host. The expected synergistic effect was calculated by multiplying phage alone reduction with the appropriate antibiotic alone challenge. For frequency of lysogenization characterization, 20 survivors for phage and all phage + antibiotic challenged were streaked purified thrice on 1% LB, and lysogen detection was performed as described below.

### Overnight challenge phage quantification

For each survivor quantification assay performed, half the volume after the 18-h challenge was filtered using a Millipore MultiScreen_HTS_ vacuum manifold (Catalog MSVMHTS00, Darmstadt, Germany) vacuum manifold and Millipore Sigma MultiScreen_HTS_ High-Volume 96-well 0.45-micron filter plate (Catalog MVHVN4525, Darmstadt, Germany). Phages in the filtrates were quantified using a standard plaque assay using PA14 as the host.

### Lysogen isolation and detection

Spot tests of phage Flora, Zilla, Nox, Drowsy, Meadow, Hali, and Cinder were carried out on PA14 and phage Flora, Nox, Meadow, Hali, and Cinder on *P. aeruginosa* clinical strain C0400. All lysates were previously amplified on host PA14 in broth. Regrowth on the plate from the phage challenge was streaked out onto 1% LB agar plate. Twenty colonies for each phage-host pair were streak purified three times and inoculated in 1 mL LB broth overnight in a deep 96-well plate. Wild type culture, not exposed to the phages, and LB broth were added as control. The plates were incubated for 18–24 h at 37°C with no agitation. The following day, the cultures were stamped on an agar overlay of either PA14 or C0400, depending on the original host used for the spot test, using a disposable pin replicator. Cultures that resulted in the clearing of the wild type host were classified as lysogen. The frequency of lysogeny was calculated as the percent of the total number of survivors that were characterized as lysogens for each phage-host pairing. One lysogen for each phage-host pair was randomly selected for ciprofloxacin induction.

### Antibiotic induction

The same-day culture of the wild type host and the lysogen were grown in the presence of two-fold dilutions of the antibiotic, prepared in nuclease-free water, in a narrow 96-well plate in 250 µL final volume. Nuclease-free water was added to the no-antibiotic growth control. The plates were incubated for 18 h at 37°C with no agitation. The following day, the plate was filtered using a vacuum manifold with a 0.45-micron filter plate. The phages in the filtrate of the untreated host (wild type and lysogen), and the host treated with antibiotic were quantified using a spot test using the wild type as the host.

### Delay administration

The same-day PA14 culture was treated with phage Hali amplified on PA14 (MOI of 10), ciprofloxacin (two-fold dilution MIC – 1/16 MIC), and combination phage Hali + ciprofloxacin in a final volume of 250 µL in a narrow 96-well plate. To test the effects of delayed administration, either antibiotic or phage was withheld for 4 h. Co-administration was tested on the same plate for each delayed administration tested. Each condition was set up in triplicates. LB broth was used as a negative control. Growth curves were carried out using OD_600_ for 4 h at 30 min intervals using the Epoch 2 microplate spectrophotometer. For delayed antibiotics, no treatment control and phage challenge were treated with nuclease-free water. For delayed phage, LB broth was added to the no-treatment and antibiotic-alone challenge. After the addition of the delayed agent, growth in the plate was measured for another 18 h at 30-min intervals. Endpoint growth (OD_600_) was calculated relative to untreated culture and plotted as a heatmap.

### Statistical tests

Details of the statistical tests can be found in the figure legend. N denotes a biological replicate performed from an independently grown culture. Quantitative values are represented by mean ± SD. All statistical analysis was done using GraphPad Prism 8.3.0 or 10.3.0 (GraphPad Software, Inc., CA, US), with a P value of ≤0.05 is considered significant.

## Data Availability

All phage sequences are available in GenBank, with the accession numbers that follow. Busker: PQ533653; Cinder: PQ533654; Doze: PQ533655; Ember: PQ533656; Frizzy: PQ533657; Hali: PQ533658; Meadow: PQ533659; Nox: PQ533660; Paws: PQ533661; Presto: PQ533662; Slumber: PQ533663; Young: PQ533664; Yoyo: PQ533665; Zilla: PQ533666; Flora bin1: PQ533667; Flora bin2: PQ533668; Pacha bin1: PQ533669; Pacha bin2: PQ533670. All raw data used to generate figures are available in the supplemental material Excel sheet. All phages isolated in this paper are available upon request. The strains or clinical isolate library information gifted to us by the Wright and Burrows labs are the property of those labs, and requests for those materials should be directed to them.

## SUPPLEMENTAL MATERIAL

Fig. S1 mbio.02559-24-s0001.tif Additional combinations.

Fig. S2 mbio.02559-24-s0002.tif Phage Hali-C0400 interactions.

Fig. S3 mbio.02559-24-s0003.tif Predictors for tPAS.

Legends mbio.02559-24-s0004.docx Legends for Fig. S1 to S3 and supplemental tables.

Supplemental tables mbio.02559-24-s0005.xlsx Raw data tables.

ASM does not own the copyrights to Supplemental Material that may be linked to, or accessed through, an article. The authors have granted ASM a non-exclusive, world-wide license to publish the Supplemental Material files. Please contact the corresponding author directly for reuse.
