# Supplementary figures and images for: Temperate phage-antibiotic synergy is widespread—extending to Pseudomonas—but varies by phage, host strain, and antibiotic pairing
Source: mBio. 2024 Dec 20;16(2):e02559-24. doi: 10.1128/mbio.02559-24 (PMC11796409; doi:10.1128/mbio.02559-24)

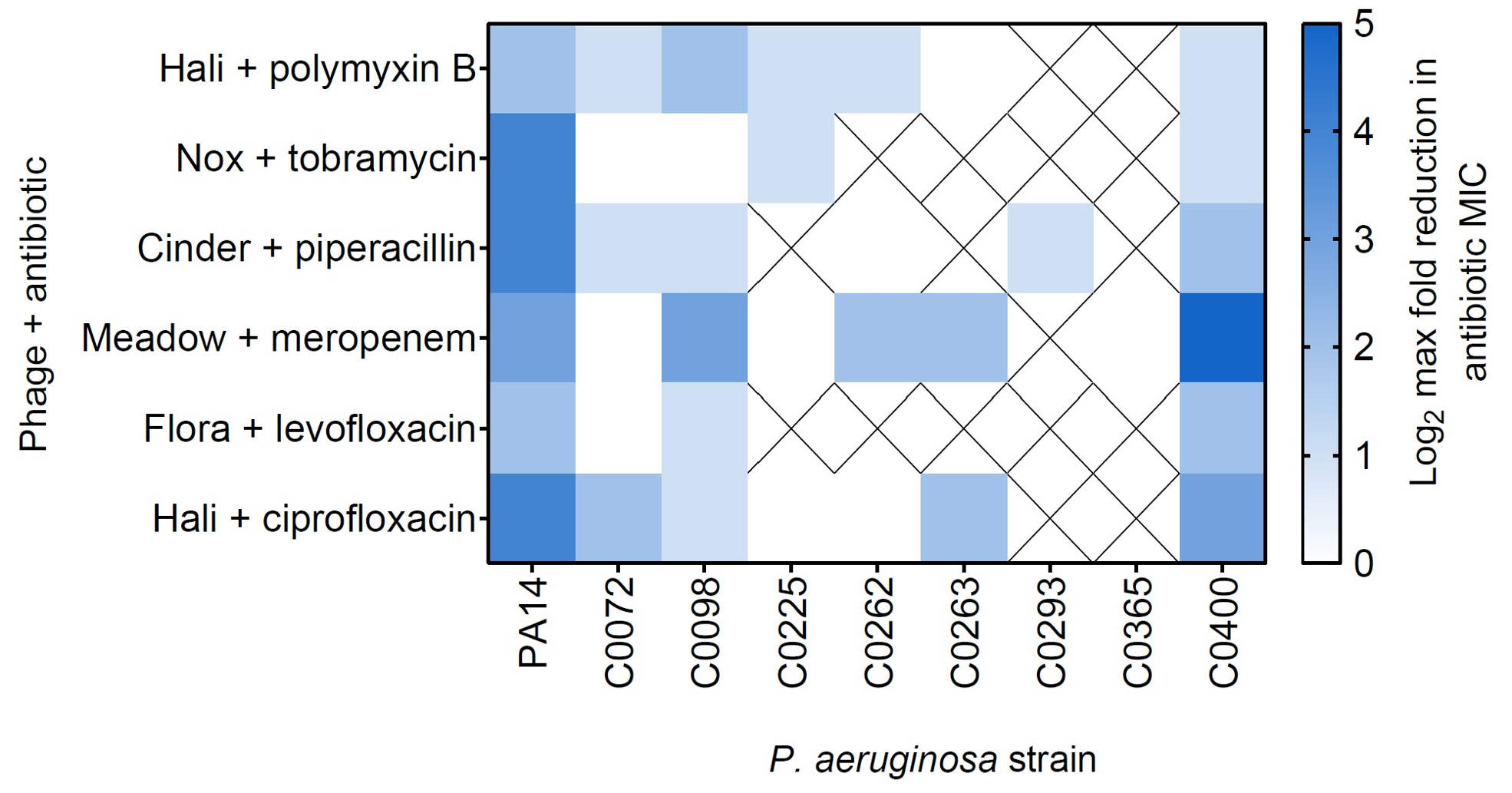

Supplement: Fig. S1 — Additional combinations. [file mbio.02559-24-s0001.tif]

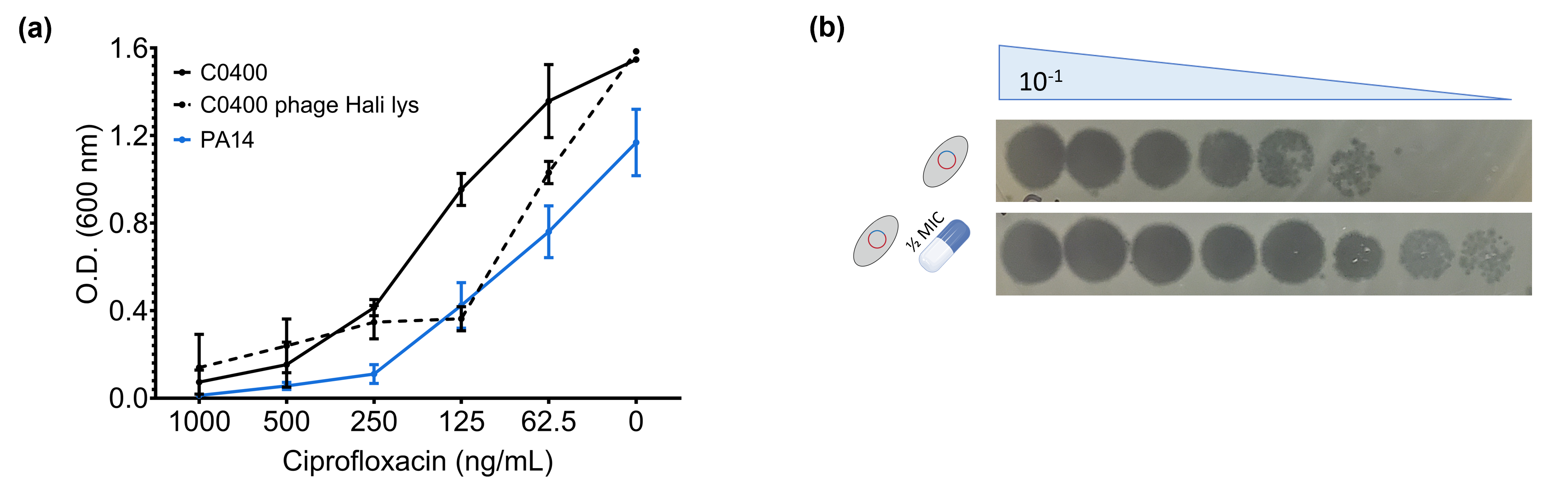

Supplement: Fig. S2 — Phage Hali-C0400 interactions. [file mbio.02559-24-s0002.tif]

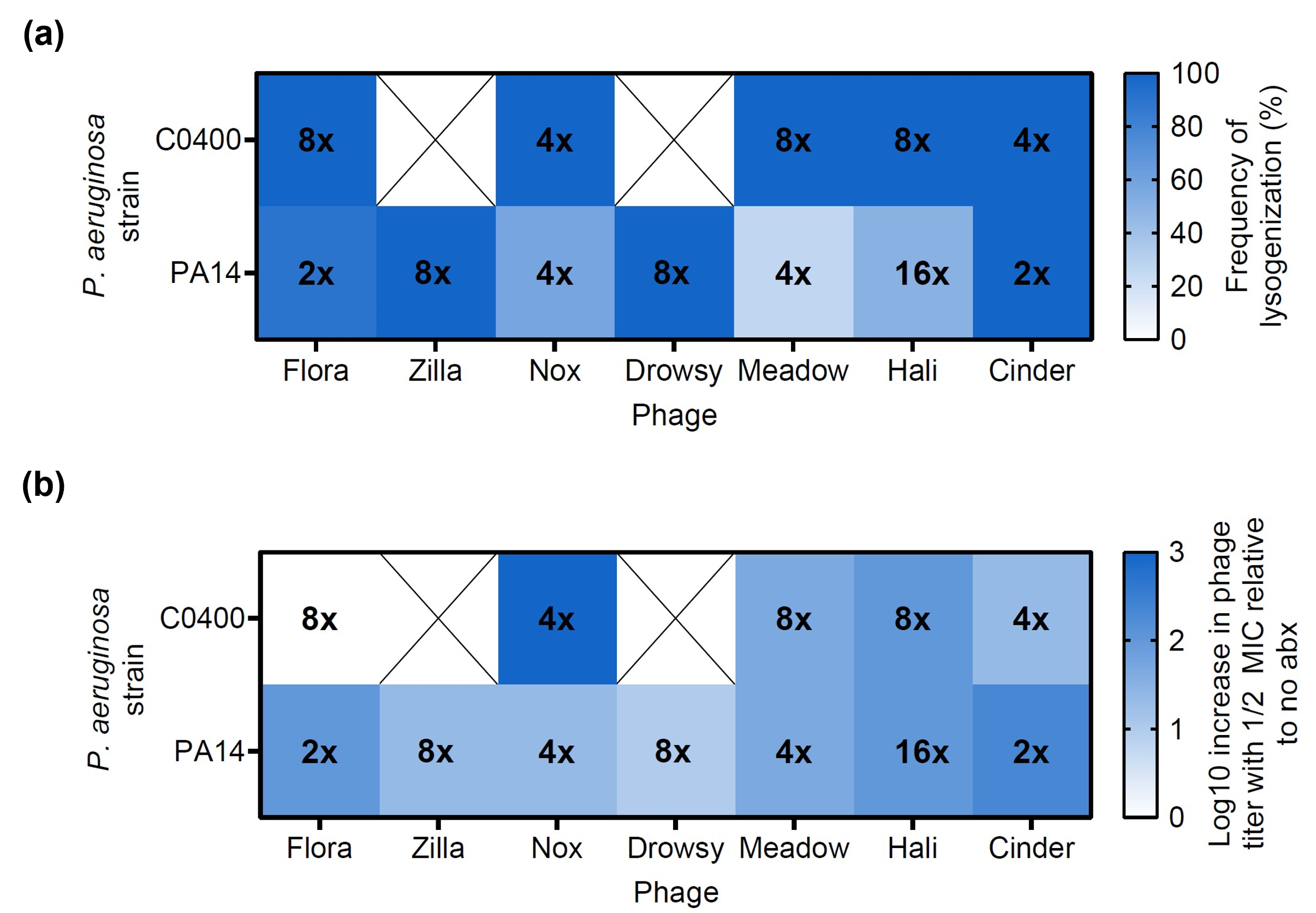

Supplement: Fig. S3 — Predictors for tPAS. [file mbio.02559-24-s0003.tif]
