# Supplementary material for: Temperate phage-antibiotic synergy is widespread—extending to Pseudomonas—but varies by phage, host strain, and antibiotic pairing
Source: mBio. 2024 Dec 20;16(2):e02559-24. doi: 10.1128/mbio.02559-24 (PMC11796409; doi:10.1128/mbio.02559-24)
Supplement: Legends — for Fig. S1 to S3 and supplemental tables. [file mbio.02559-24-s0004.docx]

**Supplementary Materials**

**SUPPLEMENTARY Figure 1.**  **PAS with temperate phages across clinical isolates.** Reduction in antibiotic MIC of multiple clinical strains with the co-administration of PA14 temperate phage. Average log_2_ maximum reduction (n=3 biological replicates, except phage Cinder + piperacillin in C0262 which is n=4) in MIC shown as heat map as determined from checkerboard assays. “X” denotes pairings that were not tested in the specified strain either due to lack of phage sensitivity or inability to obtain a high enough titer for checkerboard assay. Data represented is maximum MIC reduction regardless of phage dose (M.O.I. range of 1.25 – 40 tested in 2-fold increments).

**SUPPLEMENTARY Figure 2. C0400 phage Hali lysogen is ciprofloxacin inducible. (a)** End point growth (OD600) of C0400 (black line), C0400 phage Hali lysogen (dotted black line), and PA14 (blue line) across a range of ciprofloxacin concentrations (µg/mL) after 18 h. Data shown as mean $\pm$ SD (n=3 biological replicates, each in technical triplicates). **(b)** Representative end point phage titer of filtrates of C0400 phage Hali lysogen $\pm$½ MIC ciprofloxacin treatment.

**SUPPLEMENTARY Figure 3. Level of synergy does not correlate with frequency of lysogenization or level of induction. (a)** Frequency of lysogenization of temperate phages in host PA14 and C0400 as a heat map. Twenty survivors from phage challenge on solid media were purified and screened for the presence of the phage using a stamp test on wildtype host. Number within the colored box indicate the reduction in ciprofloxacin MIC observed in checkerboard assay. **(b)** Log_10_ average increase (n=3 biological replicate, each in single technical replicate) in phage titer when PA14 or C0400 lysogen were challenged with ½ MIC ciprofloxacin relative to no antibiotic control, represented as a heat map. X denotes combination not tested.

Supplementary Tables in Raw Data.xls : This excel file contains all data used to generate the figures in this paper, with each tab of the file named after the figure panel in which the data were used.
